# Activation of the NLRP3 Inflammasome Pathway by Prokineticin 2 in Testicular Macrophages of Uropathogenic *Escherichia coli*- Induced Orchitis

**DOI:** 10.3389/fimmu.2019.01872

**Published:** 2019-08-14

**Authors:** Ying Li, Yufang Su, Ting Zhou, Zhiyong Hu, Jiajing Wei, Wei Wang, Chunyan Liu, Huiping Zhang, Kai Zhao

**Affiliations:** ^1^Family Planning Research Institute/Reproductive Medicine Center, Tongji Medical College, Huazhong University of Science and Technology, Wuhan, China; ^2^Prenatal Diagnostic Center, People's Hospital of Guangxi Zhuang Autonomous Region, Nanning, China; ^3^Department of Gynecology and Obstetrics, Union Hospital, Tongji Medical College, Huangzhong University of Science and Technology, Wuhan, China

**Keywords:** Prokineticin 2, IL-1β, testicular macrophages, inflammasome, orchitis

## Abstract

Infections of the reproductive tract are known to contribute to testicular inflammatory impairment, leading to an increase of pro-inflammatory cytokines such as IL-1β, and a decline in sperm quality. Prokineticin 2 (PK2), a secretory protein, is closely associated with the secretion of pro-inflammatory cytokines in inflamed tissue. It was reported that increased PK2 is related to the upregulation of IL-1β, but the underlying mechanism remains elusive. Here, we illustrated that PK2 was upregulated in testicular macrophages (TM) in a rat model of uropathogenic *Escherichia coli* (UPEC) infection, which induced the activation of the NLRP3 inflammasome pathway to boost IL-1β secretion. Administration of PK2 inhibitor alleviated the inflammatory damage and suppressed IL-1β secretion. Moreover, PK2 promoted NLRP3 expression and the release of cleaved IL-1β from TM to the supernatants after the challenge with UPEC *in vitro*. IL-1β in the supernatants affected Leydig cells by suppressing the expression of genes encoding for the enzymes P450scc and P450c17, which are involved in testosterone production. Overall, we revealed that increased PK2 levels in TM in UPEC-induced orchitis may impair testosterone synthesis via the activation of the NLRP3 pathway. Our study provides a new insight into the mechanisms underlying inflammation-associated male infertility and suggests an anti-inflammatory therapeutic target for male infertility.

## Introduction

The declining in sperm quality and male infertility constitute an international concern in recent years, and infections of the reproductive tract are known to contribute to ~15% of male infertility cases. Uropathogenic *Escherichia coli* (UPEC) is estimated to be the predominant etiological agent of genitourinary infections, which can be isolated from urine and semen samples of 50 to 95% of patients (1, 2). Several investigations have proposed that the testicular impairment in an infectious process is caused by the host's immune reaction rather than by direct cytotoxic damage from the pathogen (3). Therefore, research into the immune regulation of inflammation-associated male infertility is of great significance to clinical diagnosis and treatment.

The testicular macrophages (TM) serve as the largest proportion of immune cells in the testicular interstitial space, constituting the first line of defense against pathogens. Rat TM are comprised of two distinct populations: CD68^+^-CD163^−^ (inflammatory-activated state) and CD68^+^- CD163^+^ (immunosuppressive state), and TM primarily present immunosuppressive characteristics compared with other macrophages (4). When TM were challenged with UPEC, the deficiency of IκBα degradation indicated that the NF-κB pathway was blocked, however, the phosphorylation of mitogen-activated protein (MAP) kinases was induced. Opposite results were apparent in peritoneal macrophages (5). The transcription of pro-inflammatory genes, such as interleukin-1β (IL-1β), may be triggered by MAPK activation, and IL-1β mRNA was increased in TM after UPEC infection (6).

Several studies have suggested that macrophages and IL-1β are involved in the initiation and progress of orchitis (7). IL-1β production and release occurs in two stages. It is generally recognized that NF-κB activation is the first step in evoking the synthesis of pro-IL-1β. Certain investigations suggested that MAPKs are also involved in this process (8). The second step is the inflammasome assembly, which is composed of a cytoplasmic sensor molecule, such as PYD domain-containing protein 3 (NLRP3), the adaptor protein [apoptosis-associated speck-like protein containing caspase recruitment domain (ASC)], and the effecter protein (pro-caspase-1). NLRP3 and ASC facilitate pro-caspase-1 cleavage to form the active complex, which triggers pro-IL-1β cleavage into the mature IL-1β (9). In the testis, crosstalk between Toll-like receptors and NOD-like receptors converge in the activation of the NF-κB and NLRP3 pathways, leading to IL-1β secretion from Sertoli cells (10). Moreover, NLRP3, ASC, caspase-1, and IL-1β were shown to be significantly elevated in somatic non-immune cells (Sertoli cells and peritubular cells) in a mouse model of infertility characterized by inflammation and impaired spermatogenesis (11). However, until now, no studies have concentrated on the role of NLRP3 in TM.

Prokineticin 2 (PK2), a small secretory protein, binds with its specific receptors, to mediate signal transduction to promote an inflammatory response. PK2 is expressed in peripheral blood mononuclear cells and abundantly expressed in infiltrating neutrophils and macrophages in inflamed tissues, recruiting infiltrated leukocytes during the inflammatory process (12, 13). PK2 is a central player that participates in psoriasis by promoting IL-1β expression in keratinocytes and macrophages to induce inflammatory cascades and the hyperproliferation of keratinocytes (14). Furthermore, PK2 was shown to promote lipopolysaccharides (LPS)-induced IL-1β production in peritoneal macrophages *in vitro* (15). However, the complete mechanism is unclear.

Considering the potential relationship between PK2 and IL-1β, we inferred that PK2 is closely associated with the regulation of the inflammatory process during UPEC-induced orchitis. The present study focused on determining the effect of PK2 on IL-1β secretion in TM during UPEC infection and uncovering the underlying molecular mechanisms which, ultimately, impair male fertility.

## Materials and Methods

### Animals

Adult male Wistar rats (8–10 weeks of age) were purchased from the Animal Center of Tongji Medical College. The animals were kept at 22°C with a 12 h light/dark cycle and fed with standard food pellets and water *ad libitum*. This study was performed in strict accordance with the approved guidelines from the Institutional Animal Care and Use Committee of Tongji Medical College, Huazhong University of Science and Technology (NO.2018_S808).

## Propagation of Bacteria

The UPEC strain CFT073 (NCBI: AE014075, NC_004431) characterized by Welch et al. was used (16). CFT073 fresh cultures were shaken in LB medium and grown to the early exponential phase (OD600 = 0.5–1.0). The bacterial cultures were centrifuged at 4,500× g for 8 min at room temperature. The pellets were washed with PBS and stored in DMEM/F12. A multiplicity of infection (MOI) of 20 was applied and confirmed by calculating the colony-forming units after serial dilution.

### Animal Treatment

UPEC-induced experimental orchitis was elicited in male Wistar rats as previously described (17). Under general anesthesia, the testes, epididymis, and vas deferens were exposed. Approximately 4 × 10^6^ bacterial cells in 50 μL of a UPEC saline suspension were injected into the vas deferens proximal to the cauda epididymis. Sham operated rats were injected with the same volume of saline. A ligation was made at the injection site to prevent the infection from spreading. PKR-A[(3R)-1-(4-Fluoro-3-methoxybenzyl)-N-(9-chloro-3,4-dihydro-2H-1,5-benzodioxepin-7-ylmethyl)-N-isobutylpyrrolidine-3-carboxamide)] was kindly donated by Prof. Qunyong Zhou (University of California, Irvine, USA). PKR-A was dissolved in DMSO (Sigma, USA) and diluted with PBS to adjust the concentration of DMSO to 5%. On day 1 post-infection, the UPEC-infected rats were injected with either 20 mg/kg of PKR-A or 5% DMSO into the testes every 2 days during the experiment. The sham operated rats received 5% DMSO and the rats were treated with PKR-A alone as a control.

### Sample Collection

The animals were sacrificed on day 7 post-operation. For the histopathological assessment, the testes were removed and fixed with Bouin's fluid to prepare paraffin sections to be stained with hematoxylin and eosin. To collect the testicular interstitial fluid, a small incision was made in the distal end of the testicular capsule, avoiding injury of the seminiferous tubule. Subsequently, testes were suspended in a tube at 4°C overnight. After centrifugation, the cellular contaminants were discarded.

### Sperm Preparation and Analysis

The epididymis was removed and incised into small pieces in Hams F10 medium and incubated at 37°C for 30 min to allow the spermatozoa to swim out. A drop of sperm sample was placed on a slide, and 200 spermatozoa were analyzed using light microscopy according to the criteria of the World Health Organization manual (18). Sperms of Grade A (fast progressive) and Grade B (slow progressive) were identified as progressive motility.

### Testicular Histopathology

The testes were immersed in Bouin's fluid at 4°C overnight and then embedded in paraffin. Then, the continuous paraffin sections were dewaxed with xylene and stained with hematoxylin-eosin (H&E) to detect testicular histopathology.

### Cell Isolation

Testicular macrophages and Leydig cells were isolated from adult male Wistar rats (6, 19). The animals were sacrificed by isofluran inhalation. The testes were decapsulated and digested with 1 mg/mL collagenase I (Sigma, USA) at 34°C for 15 min. After the digestion was terminated, the product was filtered to separate the interstitial cells, and seminiferous tubules.

After centrifugation and resuspension, the interstitial cells were cultured with DMEM/F12 (Life Technologies, USA). After 1 h, the non-adherent cells were removed by rinsing with the medium. The adherent interstitial cells comprised primarily macrophages, estimated at 80 to 90% by flow cytometryusing the mouse anti-CD68 monoclonal antibody (Bio-Rad, USA).

For the isolation of Leydig cells, the interstitial cells were centrifuged, resuspended, and then purified on a discontinuous four-layer Percoll (GE, USA) density gradient (21, 26, 37, and 60%). The gradient was centrifuged and the interphase between 37 and 60% was collected. The purity of Leydig cells was determined by a positive cytochemical reaction for 3β-HSD (Sigma, USA). Purity of TM and LC was 80–90%.

### Cell Treatments

For the infection of the cells, TM were primed with 1 μg/mL LPS (Sigma, USA) for 4 h or untreated, and then infected with UPEC (MOI = 20) with or without PK2 (Peprotech, USA) for 2 h. In the inhibitory experiments, LPS-primed TM were pretreated with NLRP3 inhibitor MCC950 (10 μM, Selleck Chemicals, USA) or caspase-1 inhibitor VX-765 (50 μM, Selleck Chemicals, USA) for 1 h, and then infected with UPEC (MOI = 20) with or without PK2 for 2 h.

For the detection of caspase-1 activity, TM were not pretreated with LPS. TM were treated with UPEC for 2 h in the presence or absence of PK2 and PKR-A(0.8 μM).

To disrupt testosterone production, recombinant IL-1β protein (R&D Systems, USA) was added at various concentrations (1, 10, 100 nM) to the conditioned medium in which Leydig cells were growing for 24 h. Moreover, the conditioned medium from TM with various treatments was collected and filtered and then co-cultured with Leydig cells for 24 h. The control group included Leydig cells were treated with TM culture supernatant which are not treated with LPS and UPEC etc. The anti-IL-1β antibody (10 μg/mL) was used to block IL-1β activity.

### Real-Time RT-PCR

Total RNA was isolated from the TM using TRIzol reagent (Invitrogen, USA). The reverse transcription was performed using the PrimeScript™ RT reagent Kit (Takara, Japan). PCR was performed in a 20 μL reaction mixture including 2 μL of cDNA, 0.8 μL of the forward primers and 0.8 μL of reverse primers (Table 1), and 10 μL of SYBR Green PCR MasterMix (Takara, Japan). Relative expression levels of the target genes were normalized with those of the controls using the 2^−ΔΔCt^ method.

**Table 1:** Real-time PCR primer sequences.

**Gene**	**Primer sequence**	**Length (bp)**
PK2	FP:5′-CAAGGACTCTCAGTGTGGA-3′	128
	RP:5′-AAAATGGAACTTTCCGAGTC-3′	
StAR	FP:5′-CGTGGCTGCTCAGTATTGACCTC-3′	95
	RP:5′-CAAGTGGCTGGCGAACTCTATCTG-3′	
P450scc	FP:5′-CCGGCGGATTGCAGAACTGG-3′	156
	RP:5′-TGCTTGAGAGGCTGGAAGTTGAAG-3′	
P450c17	FP:5′-GGACCAGCCAGATCAGTTCATGC-3′	164
	RP:5′-GCAGTAGCAAGGCCGTGAAGAC-3′	
17β-HSD	FP:5′-AGCGGTTTGTGGAGAAGTAGC-3′	114
	RP:5′-GTGGTTATGAGCAAGCCCTGAG-3′	
3β-HSD	FP:5′-ACCGCTGCTGTCATTGATGTCTC-3′	123
	RP:5′-GTAGATGAAGGCTGGCACACTGG-3′	
β-actin	FP:5′-GAGAGGGAAATCGTGCGT-3′	93
	RP:5′-GGAGGAAGAGGATGCGG-3′	

### Western Blot

The supernatants were added to 4 times the volume of pre-cooled acetone, incubated at −20°C overnight, and then centrifuged at 10,000 rpm for 10 min. The precipitate was washed with pre-cooled acetone to acquire the concentrated supernatants. The concentrated supernatants and cells were lysed on ice with RIPA buffer. Subsequently, 20 to 40 μg of protein were electrophoresed on an 8 to 12% sodium dodecyl sulfate-polyacrylamide gel and transferred to a nitrocellulose membrane. The membranes were blocked with 5% non-fat milk for 1 h in TBS and subsequently incubated with the following primary antibodies: goat anti-IL-1β polyclonal antibody (1:1,000, R&D Systems, USA), rabbit anti-NLRP3 polyclonal antibody (1:500, Novus, USA), mouse anti-caspase-1 monoclonal antibody (1:500, Novus, USA), mouse anti-β-actin polyclonal antibody (1:500, Boster, China), and mouse anti-PK2 monoclonal antibody (1:2,000, donated by Prof. Qunyong Zhou). The protein bands were detected using ECL (Pierce, USA).

## ELISA

The levels of PK2 and IL-1β in the testicular interstitial fluid and cell supernatants were quantified using specific sandwich ELISA following the manufacturer's instructions. PK2 was measured using an ELISA Kit from CUSABIO (China). IL-1β levels were evaluated using an ELISA Kit from R&D Systems (USA).

### Immunofluorescence

Cells seeded in a cover glass were washed with PBS and then fixed with pre-cooled 4% formaldehyde. Subsequently, the cells were blocked with 5% normal goat serum at room temperature for 1 h and then incubated with the rabbit anti-PK2 polyclonal antibody (1:200, Abcam, USA) and mouse anti-CD68 monoclonal antibody (1:200, Abcam, USA) overnight. After washing twice with PBS, the cells were incubated with the appropriate secondary antibodies: donkey anti-mouse IgG H&L (Alexa Fluor^®^ 647) (1:500, Abcam, USA) and goat anti-rabbit IgG H&L (FITC) (1:500, Abcam, USA) at room temperature for 1 h. The cell nuclei were stained with DAPI (biofroxx, Germany).

### Flow Cytometric Analysis

For the flow cytometric analyses, 350 μL of interstitial single-cell suspensions (approximately 1 × 10^6^ cells) were incubated with 2 μL of mouse anti-CD45-PE/Cy7 (BioLegend, USA) and 2 μL of mouse anti-CD163-Alexa Fluor 647 monoclonal antibody (Bio-Rad, USA). After centrifugation, the pellets were resuspended and incubated in fixation/permeabilization solution (BD, USA). Subsequently, the cells were incubated with 2 μL of mouse anti-CD68-Alexa Fluor 488 (Bio-Rad, USA). After excluding cell aggregates and doublets based on side scatter A (SSC-A) vs. SSC-H plot, CD45^+^ cells were analyzed to identify the testicular macrophage population by CD68 and CD163. Flow cytometry analysis was performed by using a flow cytometer (BD LSR II, USA), and data were analyzed with FlowJo software version X.

### Caspase-1 Activity Assay

A Caspase-1 Activity Kit (BioVision, Mountain View, CA, USA) was used to evaluate caspase-1 activity. The yellow formazan product p-nitroaniline (pNA) was converted from the labeled substrate acetyl-Tyr-Val-Ala-Asp *p*-nitroanilide (Ac-YVAD-*p*NA) by activated caspase-1. Cell lysates were incubated with 5 μL of Ac-YVAD-*p*NA (4 mM) at 37°C for 1 h. The absorbance values of *p*NA were detected at 405 nm. The product of pNA reflected the activation of caspase-1.

### Testosterone Detection

For the chemiluminescent immunoassay, the serum levels of testosterone were measured using a chemiluminescent immunoassay kit (Cloud clone, China) following the manufacturer's protocols. According to the standard curve of known concentrations, the testosterone level was extrapolated.

For the liquid chromatography-mass spectrometry (LC-MS), 200 μL sample was taken, and pre-cooled 800 μL of methanol/acetonitrile (1:1) was added to precipitate the protein, then centrifuged at 14,000 g for 4 min at 4°C. Then, the supernatants was taken and dried under vacuum. Then, 100 μL acetonitrile-water (1:1) was added. Afterthat, the fluid was centrifuged at 14,000 g for 15 min at 4°C. The treated supernatants was analyzed in liquid chromatograph LC-30A (SHIMADZU, Japan) and 4,500 QQQ mass spectrometer (AB Sciex, American).

### Statistical Analysis

Statistical package for social science (SPSS) 18.0 and GraphPad Prism software 5.0 were used to conduct the statistical analyses. Data are presented as the mean ± standard error of the mean (SEM). Differences between two groups were analyzed by an unpaired *t*-test. Intergroup differences in multiple groups were measured using one-way ANOVA with Tukey's HSD *post hoc* test. *P* < 0.05 were considered significant.

## Results

### PK2 Is Upregulated in TM of UPEC-Induced Rat Orchitis

**Figure 1:**
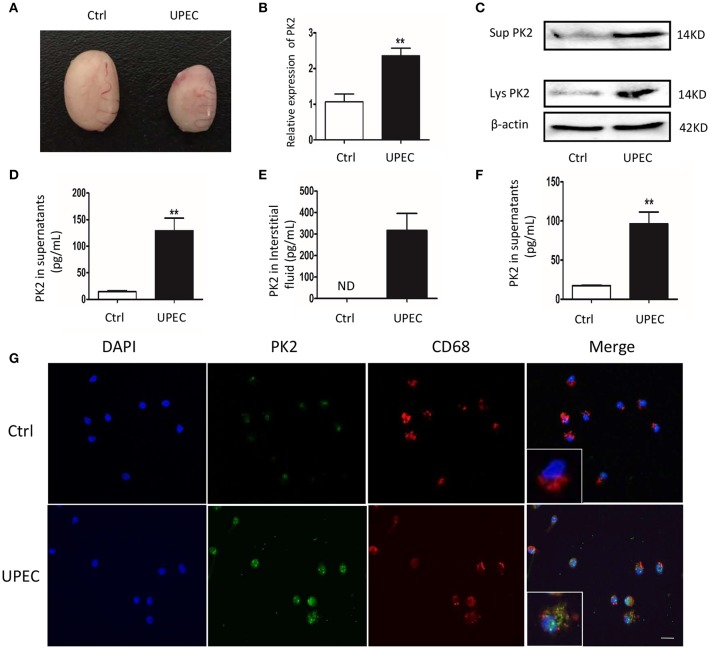
PK2 is upregulated in TM in UPEC-induced orchitis. UPEC saline suspension was separately injected into the vas deferens proximal to the cauda epididymis, which reached the testis via a retrograde infection. Sham operated rats injected with the same volume of saline served as the control. **(A)** Testicular morphology, Ctrl (left) and UPEC (right) (*n* = 5/group). **(B)** PK2 mRNA expression in TM was detected by PCR (*n* = 5/group). **(C)** The cells were isolated from control and UPEC-treated rat testes, and then the medium was changed after the cells adhered to the wall for 1 h. After a further culture for 2 h, TM and the supernatants were collected. The protein level of PK2 in TM and the supernatant was analyzed by western blot. **(D)** The PK2 protein level in the supernatants of TM isolated from rats was analyzed by ELISA (*n* = 5/group). **(E)** The protein level of PK2 in the testicular interstitial fluid was determined by ELISA (*n* = 5/group). **(F)** TM were sham treated or UPEC infected (MOI, 20) for 2 h *in vitro*. PK2 level expression in the supernatants was determined by ELISA (*n* = 5/group). **(G)** TM were isolated from the rat testes of two groups. PK2 expression in TM was detected by immunofluorescence. Scale bar 50 μm. Ctrl, control; Lys, cell lysates, Sup, supernatants. **P < 0.01 (*t*-test).

To explore the role of PK2 in UPEC-induced orchitis, a rat model was established according to a previously described method (17). Seven days after the treatment, the testes exhibited characteristics including a smaller size, various degrees of swelling, and a softer texture compared with the control rats (Figure 1A). Given that the invasion and location of UPEC were detected in the testicular interstitium (17), we focused on TM, which are the most abundant immune cells in the testicular interstitium. PK2 mRNA expression was upregulated in TM isolated from the UPEC-infected rats (Figure 1B). In addition, PK2 protein level was obviously elevated in cell lysates, as well as in TM supernatants of the UPEC-infected group (Figure 1C, Supplementary Figures S1, S9). The level of PK2 in the supernatants of the UPEC-infected group was remarkably higher compared with the control group, according to the quantitative analysis (Figure 1D). The micro-environmental conditions were evaluated in the interstitial fluid surrounding the TM, and the protein level of PK2 in the interstitial fluid was measured by ELISA. The expression of PK2 was not found in normal interstitial fluid, whereas it was dramatically increased in the UPEC-infected group (Figure 1E). Next, the capacity of UPEC to trigger PK2 release *in vitro* was also determined. Normal TM secreted little PK2, while UPEC promoted the release of PK2 (Figure 1F). Subsequently, the location of PK2 in the TM was identified by the expression of the rat macrophage surface receptor marker CD68 in the different groups. PK2 was observed in the nucleus in the control group. However, UPEC infection induced PK2 to diffuse throughout the cytoplasm, indicating that secretory PK2 protein was engaged in an efficient production and release process (Figure 1G). In summary, TM were induced to express pro-inflammatory PK2 in UPEC-infected rats, which abundantly accumulated in the cytoplasm and was released into the extracellular fluid to a greater extent compared with the control.

### PKR-A Alleviates UPEC-Induced Testicular Inflammation

**Figure 2:**
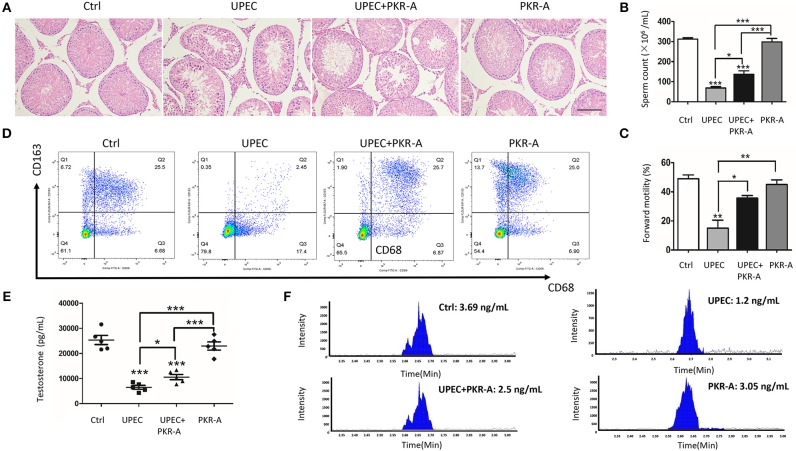
PK2 impairs testicular histological structure, testosterone synthesis, and semen quality. The UPEC-infected rats were injected with either 20 mg/kg of PKR-A or 5% DMSO into the testes every 2 days during the experiment. The sham operated rats received 5% DMSO and the rats were treated with PKR-A alone as a control. **(A)** Testicular histological changes were detected by H&E staining (*n* = 5/group). **(B)** Sperm count was analyzed (*n* = 5/group). **(C)** Sperm forward motility was analyzed (*n* = 5/group). **(D)** In the flow cytometry examination, the total leukocyte population of testicular interstitial cells was marked by the expression of CD45, and gated for the identification of rat macrophage subsets by the surface markers of CD68 with or without CD163(*n* = 5/group). **(E)** The testosterone level in the serum was detected by chemiluminescent immunoassay (*n* = 5/group). **(F)** The testosterone level in the serum was detected by LC-MS. Scale bar 100 μm. Ctrl, control. *P < 0.05, **P < 0.01, ***P < 0.001 (one-way ANOVA).

Considering the pro-inflammatory role of PK2, its effects on the progression of the inflammatory response and male fertility were investigated. PAR-A, an inhibitor of PK2, was administered via injection to UPEC-infected rat testes to suppress PK2 activity. After UPEC infection, inflammatory damage of testicular tissue and abnormal morphology of spermatogenic cells in tubules were observed. However, after PKR-A treatment, inflammation was alleviated, and the damage to germ cells damage was milder (Figure 2A). Furthermore, UPEC exerted a negative influence on total sperm count and forward motility, while the UPEC+PKR-A group exhibited a partial recovery of these factors (Figures 2B,C). In the UPEC-infected group, the proportion of CD68^+^- CD163^+^ macrophages with immunosuppressive effects was decreased compared to the control group. However, this change was reversed after PKR-A treatment (Figure 2D). UPEC reduced testosterone production in the serum, while the UPEC+PKR-A group exhibited a partial recovery (Figure 2E). The results of LC-MS further verified the trend of testosterone production in different treatment groups (Figure 2F). Taken together, our findings suggested that PK2 promoted inflammation in the testis, which impaired male reproduction, however, the PK2 antagonist PKR-A alleviated the inflammation and facilitated the recovery of sperm count, forward motility and testosterone production. The rats treated with PKR-A alone had no significant changes in testicular histomorphology and parameters related to male reproduction.

### Inhibition of PK2 Activity Decreases IL-1β Secretion

**Figure 3:**
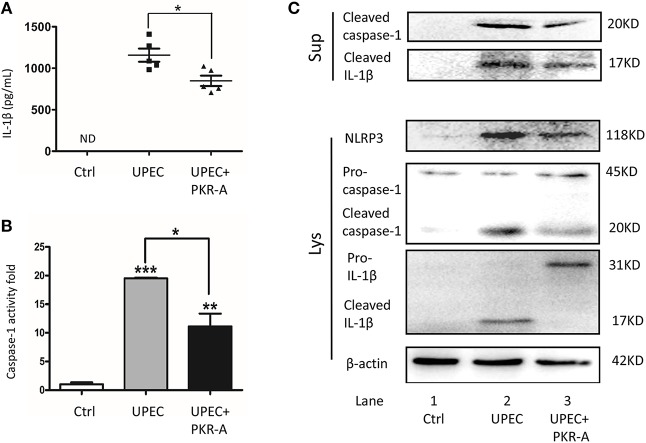
Inhibition of PK2 results in decreased IL-1β secretion. The UPEC-infected rats were injected with either 20 mg/kg of PKR-A or 5% DMSO into the testes every 2 days during the experiment. The sham operated rats received 5% DMSO as a control. TM were isolated from the three groups. The supernatants were collected after the isolated TM were further cultured for 2 h. **(A)** IL-1β secretion of the testicular interstitial fluid was analyzed by ELISA (*n* = 5/group). **(B)** Whole-cell lysates were analyzed by caspase-1 activity assay to determine the cleaved-caspase-1 activity (*n* = 3/group). **(C)** Concentrated supernatants were analyzed by western blot with antibodies against IL-1β, caspase-1. Whole-cell lysates were analyzed by western blot with antibodies against IL-1β, caspase-1, NLRP3, and β-actin. Ctrl, control; ND, no detected; Lys, cell lysates, Sup, supernatants. *P < 0.05, **P < 0.01, ***P < 0.001 (one-way ANOVA).

To explore the potential involvement of PK2 in IL-1β secretion, IL-1β-associated pathways were investigated. UPEC triggered robust IL-1β release into the interstitial fluid, however, this response was greatly reduced after PKR-A treatment (Figure 3A). IL-1β release is considered to be a marker for inflammasome activation, thus we further investigated other associated components of the inflammasome pathway. A similar trend was observed for cleaved caspase-1 activity: caspase-1 cleavage was elicited in TM from rats infected with UPEC, but this effect was weakened in the PKR-A-treated group (Figure 3B). To further clarify the specific mechanism of inflammasome activation, the different expression patterns of caspase-1 and IL-1β in TM, and the supernatants were assessed by western blot. UPEC induced a significant increase of cleaved IL-1β and cleaved caspase-1 release from the TM into the supernatants compared with the uninfected rats, but this release was decreased by PKR-A treatment. In TM, the protein level of NLRP3, cleaved caspase-1, and cleaved IL-1β were markedly increased in the UPEC-infected group, but this increase was attenuated in the UPEC+PKR-A group. UPEC promoted the cleavage of pro-IL-1β to form mature IL-1β to be released, thus the level of the pro-IL-1β protein was decreased in the infected group. Interestingly, after the PKR-A treatment, pro-IL-1β expression remained at a high level in the TM (Figure 3C, Supplementary Figures S2–S6). Taken together, our findings suggested that the NLRP3 inflammasome pathway was activated in TM after UPEC infection *in vivo*, leading to the cleavage of pro-IL-1β to IL-1β. In addition, PK2 was involved in the NLRP3 inflammasome activation.

### PK2 Promotes the Activation of the NLRP3 Inflammasome Pathway

**Figure 4:**
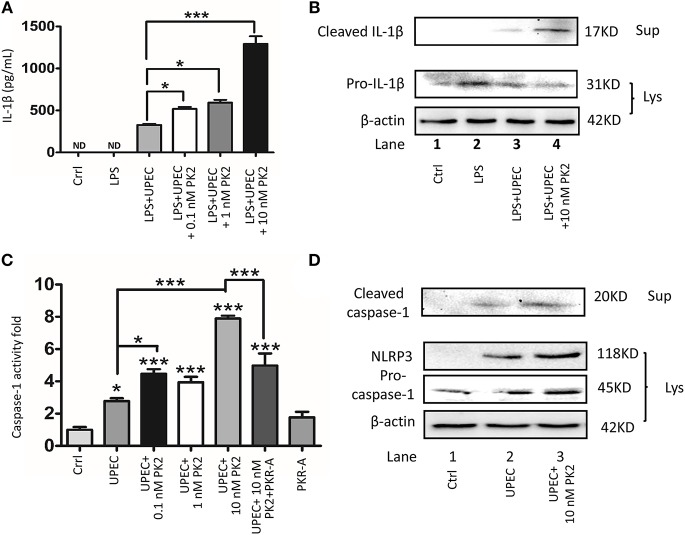
PK2 promotes the NLRP3 pathway. **(A)** TM were primed with 1 μg/mL LPS for 4 h and then infected with UPEC (MOI 20) with or without various concentrations of PK2 (0.1 nM, 1 nM, and 10 nM) for 2 h. IL-1β secretion in the supernatants was analyzed by ELISA. **(B)** TM and the supernatants were treated as described above. Whole-cell lysates and concentrated supernatants were analyzed by western blot with against IL-1β and β-actin. **(C)** TM were infected with UPEC (MOI = 20) with or without PK2 for 2 h. Whole-cell lysates were analyzed by caspase-1 activity assay. **(D)** TM were treated as described above. Whole-cell lysates were analyzed by western blot with antibodies against caspase-1 and concentrated supernatants were analyzed by western blot with antibodies against caspase-1, NLRP3, and β-actin. Ctrl, control; LPS, lipopolysaccharide; ND, no detected; Lys, cell lysates, Sup, supernatants. *P < 0.05, ***P < 0.001 (one-way ANOVA).

PK2 and its receptor follow the G-protein-coupled receptor signaling pattern, activate the PK2 transcription, release the functional PK2 protein from the cytoplasm to the extracellular milieu, and further activate this pathway (20). Given that UPEC directly triggered PK2 release, and the interstitial fluid contained a high concentration of PK2 in the UPEC-infected rats, ectogenic PK2 was applied to mimic the TM milieu to boost this positive feedback process to illuminate the effects of PK2 on the NLRP3 inflammasome pathway *in vitro*. Moreover, LPS pretreatment was administered to promote pro-IL-1β levels, facilitating the evaluation of IL-1β release as a hallmark for inflammasome activation with minimal interference by UPEC (21). It was found that IL-1β secretion in the supernatants was increased by UPEC with LPS priming. The additional PK2 induced IL-1β secretion in a dose-dependent manner (Figure 4A). As illustrated in the western blot results, cleaved IL-1β in the supernatants was higher in the UPEC with or without PK2 group compared to the control or LPS alone treatment group (Figure 4B, Supplementary Figures S6–S8). Correspondingly, a significant increase in cleaved caspase-1 activity occurred after UPEC infection, and higher cleaved caspae-1 production was further promoted by the additional PK2 (Figure 4C). Cleaved caspase-1 in the supernatants and NLRP3 expression in TM were strongly upregulated when the cells were challenged with UPEC with or without PK2, compared unstimulated cells (Figure 4D, Supplementary Figures S4, S7–S9). Taken together, these findings indicate that PK2 augments the effects of UPEC on the activation of the NLRP3 inflammasome pathway *in vitro*.

### PK2-Mediated IL-1β Secretion Is Dependent Upon the NLRP3 Pathway

**Figure 5:**
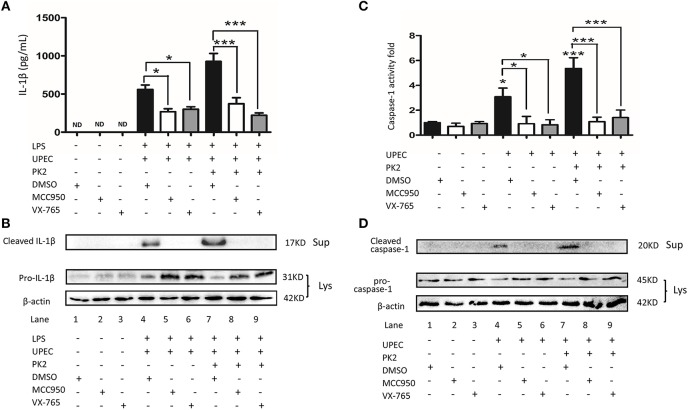
The UPEC-mediated NLRP3 inflammasome activation is suppressed by NLRP3 and caspase-1 inhibition. **(A)** TM were primed with 1 μg/mL LPS for 4 h or left untreated, pretreated for 1 h with the NLRP3 inhibitor (10 μM) or caspase-1 inhibitor (50 μM), and subsequently infected with UPEC (MOI = 20) with or without 10 nM PK2 for 2 h. The supernatants were collected at 2 h post-infection and analyzed for IL-1β release using ELISA. **(B)** TM were treated as described above, cell lysates as well as the supernatants were analyzed by western blot for IL-1β, and β-actin. **(C)** TM were pretreated for 1 h with the NLRP3 inhibitor (10 μM) or caspase-1 inhibitor (50 μM) and subsequently infected with UPEC (MOI = 20) with or without 10 nM PK2 for 2 h. Whole-cell lysates were analyzed by caspase-1 activity assay. **(D)** TM were treated as described above. Whole-cell lysates and concentrated supernatants were analyzed by western blot with antibodies against caspase 1, β-actin. Ctrl, control; LPS, lipopolysaccharide; DMSO, dimethylsulfoxide; ND, no detected; Lys, cell lysates, Sup, supernatants. *P < 0.05, ***P < 0.001 (one-way ANOVA).

To further verify the involvement of the NLRP3 pathway in TM challenged by UPEC with or without PK2, MCC950 (NLRP3 inhibitor), and VX-765 (caspase-1 inhibitor) were used. In LPS-primed TM, MCC950, and VX-765 significantly suppressed the UPEC-induced IL-1β release in the supernatants, and additional PK2 did not reverse this trend (Figure 5A). Similarly, in LPS-primed TM, cleaved IL-1β was detected in the supernatants after UPEC infection with or without PK2 treatment, but the presence of cleaved IL-1β in the supernatants was eliminated by the inhibitors (Figure 5B, Supplementary Figures S10–S12). Cleaved caspase-1 activity was stimulated by UPEC with or without PK2, but this activation was substantially depressed by MCC950, and VX-765 (Figure 5C). After the intervention with inhibitors, cleaved caspase-1 was not observed in the supernatants after UPEC infection with or without PK2 (Figure 5D, Supplementary Figures S4, S13, S14). Collectively, these data further confirmed that the NLRP3 inflammasome pathway is involved in PK2-mediated IL-1β secretion in TM.

### IL-1β Inhibits Testosterone Production by Suppressing P450scc and P450c17 Expression

**Figure 6:**
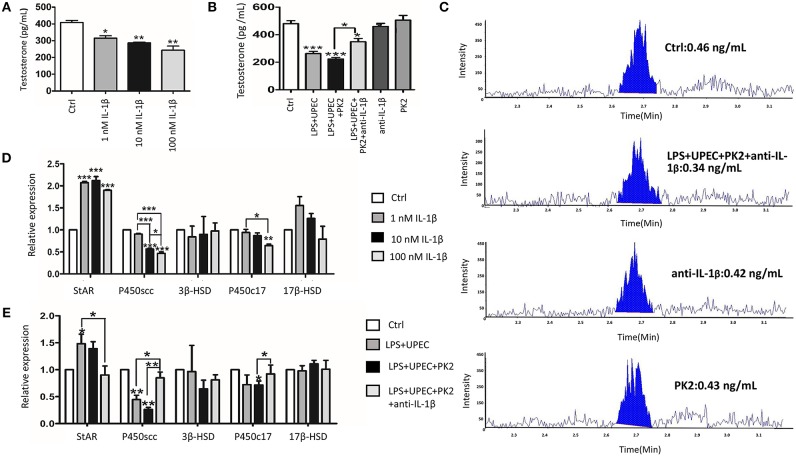
IL-1β inhibits testosterone production by repressing the expression of P450scc and P450c17. **(A)** Testosterone produced by Leydig cells under the stimulation of various concentrations of IL-1β (1 nM, 10 nM, and 100 nM) for 24 h. **(B)** The conditioned medium from TM without treatment (control) or with indicated treatments was collected, filtered, and then co-cultured with Leydig cells for 24 h. **(C)** The testosterone production by Leydig cells was detected by LC-MS. The content of IL-1β in the supernatants of some treatment groups (100 nM IL-1β, LPS+UPEC, LPS+UPEC+10 nM PK2) is lower than the limit of detection, the corresponding results are not shown. **(D)** The mRNA expression of StAR, P450scc, P450c17, 3β-HSD, and 17β-HSD in Leydig cells treated with various concentrations of IL1β (1 nM, 10 nM, and 100 nM) for 24 h was detected by PCR. **(E)** The mRNA expression of StAR, P450scc, P450c17, 3β-HSD, and 17β-HSD in Leydig cells treated with TM-condition medium for 24 h was detected by PCR. Ctrl, control. *P < 0.05, **P < 0.01, ***P < 0.001 (one-way ANOVA).

TM are structurally coupled with androgen-producing Leydig cells in the testicular interstitial space to constitute an immune-endocrine compartment. To determine whether IL-1β directly affects Leydig cells steroidogenesis, primary Leydig cells were treated with various concentrations of IL-1β (1 nM, 10 nM, and 100 nM). The results revealed that, indeed, IL-1β directly inhibited testosterone biosynthesis in a dose-dependent manner (Figure 6A). Given that IL-1β has a pronounced influence on testosterone production, we hypothesized that the IL-1β secretion from TM challenged with UPEC may inhibit testosterone synthesis. The supernatants of the LPS-primed TM after UPEC infection with or without PK2 were co-cultured with primary Leydig cells. Testosterone production was shown to be decreased in Leydig cells after they were co-cultured with TM supernatants. This hypothesis was further supported by using an anti-IL-1β antibody to neutralize IL-1β, which resulted in a marked increase in testosterone synthesis (Figure 6B). The results of LC-MS indicated that testosterone can be detected in the control group, however, the content of testosterone in the supernatants of some treatment groups (100 nM IL-1β, LPS+UPEC, LPS+UPEC+10 nM PK2) is lower than the limit of detection, the corresponding results are not shown (Figure 6C). To explore the mechanism underlying the IL-1β-induced inhibition of testosterone production, the mRNA expression of enzymes that are crucial for testosterone synthesis was detected in Leydig cells stimulated with IL-1β in various concentrations. The results showed that the mRNA expression of steroidogenic acute regulatory protein (StAR) was increased, whereas cholesterol side-chain cleavage P450 (P450scc), and 17α-hydroxylase/C_17−20_ lyase (P450c17) were decreased, and no significant differences were found in the expression of 3β-hydroxysteroid dehydrogenase-Δ^4^-Δ^5^ isomerase (3β-HSD) or 17β-hydroxysteroid dehydrogenase (17β-HSD) (Figure 6D). Then, the expression of genes encoding for key enzyme were detected in Leydig cells that were co-cultured with various supernatants. The results closely resembled those of the IL-1β treatment; upregulation of StAR and downregulation of P450scc and P450c17 were observed. These alterations were repressed by the anti-IL-1β antibody (Figure 6E). Taken together, these findings indicate that IL-1β secretion from TM exerts a negative effect on testosterone production by repressing the expression of P450scc and P450c17.

## Discussion

In this study, our results suggested that UPEC infection induces testicular inflammatory responses by promoting NLRP3 inflammasome activation in TM. Interestingly, the increased PK2 in TM and the testicular interstitial fluid augments this process. Moreover, IL-1β secretion from TM suppresses testosterone synthesis, which reduces the sperm count and forward motility. We reveal a novel mechanism of PK2 in regulating the NLRP3 inflammasome pathway to aggravate inflammatory damage and reduce testosterone production, which ultimately impairs male fertility.

There is growing evidence that increased PK2 levels in inflamed tissue is closely associated with the infiltration of inflammatory cells, thus PK2 may be a link between immune cells and the development of the inflammatory response (22, 23). In animal models of paw inflammation, the severity and duration of pain were related with the upregulation of PK2 expression in granulocytes (13). The relationship between PK2 and pathogen infection has also received considerable attention. LPS and bacterial DNA directly stimulated the upregulation of PK2 in Raw 264.7 cells *in vitro* (14). Our findings were consistent with previous findings, as the upregulation of PK2 mRNA and protein was detected in TM isolated from the UPEC-infected rats. Moreover, the supernatants of TM stimulated with UPEC and the testicular interstitial fluid exhibited the increased PK2 level. Overall, we inferred that the direct stimulation of invasive UPEC in the testicular interstitium induced the increased PK2 level. Furthermore, previous studies revealed that the PK2 signaling pathway involves both autocrine and paracrine loops to participate in multiple biological processes, such as the differentiation of cardiac and renal cells (24) and tumor angiogenesis (20). Given that the upregulation of the PK2 protein in the TM cytoplasm and the extracellular milieu, we inferred that UPEC not only upregulated PK2 transcription but also promoted the release of the functional PK2 protein from the TM to the testicular interstitium to further promote PK2 production.

Innate immune responses to pathogens are related with the assembly of large protein complexes defined as inflammasomes, and the functional component of this process is the release of the pro-inflammatory cytokine IL-1β (25). Zika-induced IL-1β release was observed in human PBMCs and mouse bone marrow dendritic cells via the combining of the NS5 protein with NLRP3 (26). The bacterium *Acinetobacter baumannii* induced IL-1β secretion via the NLRP3-ASC-caspase-1 pathway, resulting in lung damage (27). Although the inflammation-related upregulation of IL-1β was previously observed in UPEC-infected TM, whether the NLRP3 inflammasome has any role in the pathogenesis was unknown. As the priming signal for IL-1β secretion, pro-IL-1β should be synthesized, which is thought to be primarily regulated in an NF-κB-dependent manner. However, recent studies have reported that the inflammasome is activated independent of NF-κB (28). Moreover, MAPK but not the NF-κB pathway was activated in TM challenged with UPEC (5, 6).

A second sequential signal, further stimulation, such as bacteria, triggers the NLRP3-ASC-caspase-1 assembly. In this study for the first time, we systematically demonstrated a significant increase in IL-1β release and cleaved caspase-1 production in UPEC-infected rat TM. NLRP3 expression was identified to be a critical hallmark for NLRP3 activation (29), and the upregulation of the NLRP3 protein was also observed in TM isolated from UPEC-infected rats compared with the uninfected group in our study. Furthermore, TM were pretreated with LPS to acquire the priming signal *in vitro* as previously mentioned (21), and further stimulation with UPEC remarkably induced the production of the NLRP3, cleaved caspase-1, and cleaved IL-1β proteins. Moreover, the NLRP3 or caspase-1 inhibitors exerted clear inhibitory effects on the release of IL-1β. Interestingly, LPS profoundly induced the synthesis of pro-IL-1β in TM, however, cleaved IL-1β could not be detected in the supernatants. LPS stimulated the TM to release pro-inflammatory cytokines, such as TNF-α, at much lower levels compared to those in peritoneal macrophages (30), thus we inferred that the immunological characteristics of TM and the deficiency of inflammasome activation upon LPS stimulation may be attributed to the absence of IL-1β.

IL-1β was not induced by PK2 in unstimulated cells, however, PK2 remarkably facilitates IL-1β release in LPS-stimulated peritoneal macrophages (15). PK2 promotes the activation of the MAPK and AIM2 signaling pathways, and IL-1β secretion in THP-1 cells, however, systematic research on the underlying mechanism remains limited (14). In the UPEC-infected model, we demonstrated that PK2, and IL-1β expression were abnormally high in TM. To further investigate the relationship between these proteins, PK2 signaling inhibitor PKR-A was applied. In the animal model of stroke, when PK2 was delivered into the lateral ventricle post-stroke, CD68^+^ cells were significantly increased. (18). PKR-A was also administered in an animal model of collagen-induced arthritis (31) and autoimmune encephalomyelitis (32). In our UPEC-infected model, IL-1β secretion and caspase-1 cleavage were decreased after PKR-A treatment. Furthermore, in the western blot analysis, pro-IL-1β was almost completely proteolytically cleaved to form the mature IL-1β in the UPEC-infected group, whereas pro-IL-1β was maintained in the PKR-A treated group, suggesting that the inflammasome effect on pro-IL-1β cleavage was repressed by PKR-A. Then, PK2 was added to TM-conditioned medium to verify the effect of PK2 on the inflammasome activation. PK2 significantly increased the expression of NLRP3, cleaved caspase-1 and cleaved IL-1β after the UPEC infection, and this activation was suppressed by MCC950, and VX-765. This is the first report that PK2 plays a critical role in triggering the release of IL-1β in TM through the NLRP3 inflammasome pathway.

The testicular interstitium, an immune-endocrine compartment, contains a significant proportion of macrophages that are structurally coupled with Leydig cells (33). Several investigations suggested that activated macrophages induce the release of pro-inflammatory cytokines, such as IL-1β, which profoundly repress the expression of genes encoding for steroidogenic enzymes in Leydig cells (34). IL-1β is not detected under normal physiological condition, and the absence of IL-1R has no effect on testosterone synthesis; IL-1R is required for IL-1-induced signal transduction in mice (35). In our study, primary Leydig cells were isolated and then cultured with various concentrations of IL-1β. The results revealed that testosterone levels, P450scc and P450c17 synthesis were reduced, which was consistent with previous data (36), however, the mRNA expression of StAR was shown to be increased in our study. The steroidogenesis process in Leydig cells includes the following two principal activities: the first step is an acute cAMP-dependent process requiring the action of the StAR. The second step is a chronic and durable stimulation, which involves the gene expression of the steroidogenic enzymes: P450scc, 3β-HSD, P450c17, and 17β-HSD (37). We inferred that IL-1β triggers an acute compensatory response of testosterone synthesis via the upregulation of StAR, and then testosterone production is repressed by inhibiting P450scc, and P450c17 in a chronic and durable process. TM-conditioned medium was co-cultured with Leydig cells to investigate the regulatory mechanism of immune-endocrine interactions (38). The conditioned medium collected from TM with various treatments was co-cultured with primary Leydig cells in our research. The results were consistent with previous experiments involving IL-1β intervention, and the suppressive effect was abolished with an anti-IL-1β neutralizing antibody, which further supported our hypothesis.

**Figure 7:**
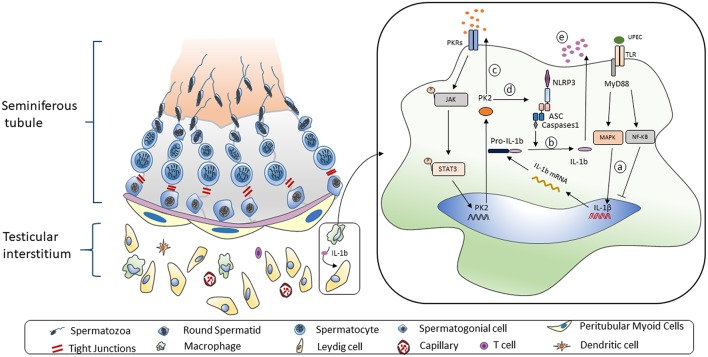
Working model of UPEC-induced NLRP3 inflammasome activation in TM. **Left:** The mammalian testis is compartmentalized to two main parts. The seminiferous tubes are composed of various developing germ cells surrounded by Sertoli cells, while the testicular interstitial space consists of androgen-producing Leydig cells, and multiple types of immune cells. A significant proportion of macrophages is structurally coupled with Leydig cells with anatomical and functional relation. **Right:** When TM challenged by UPEC a. Activation of MAPK pathway but not NF-κB pathway induces the pro-IL-1β synthesis. b. The NLRP3 inflammasome pathway is triggered to secret IL-1β. c. Biological function of TM-derived PK2 is augmented and sustained via an autocrine and paracrine manner. d. PK2 promotes NLRP3 expression and boost inflammasome pathway activation to secret IL-1β. e. IL-1β is released to testicular interstitium from TM to suppress the testosterone synthesis.

In our research, the administration of PKR-A relieved the inflammatory damage to seminiferous epithelium cells and reduced the portion of inflammatory-activated state macrophages. Our results also suggested that after PKR-A treatment, the inflammation-induced testosterone reduction was attenuated, leading to an increase in the total sperm count and forward motility. Overall, PK2 plays important roles in the pro-inflammatory process of UPEC-induced orchitis by promoting IL-1β secretion in TM. A novel mechanism through which PK2 induces the NLRP3 inflammasome activation to boost IL-1β maturation after UPEC infection is presented here. The IL-1β is released from TM to the testicular interstitium where it affects adjacent Leydig cells and inhibits testosterone synthesis, leading to the impairment of spermatogenesis and, ultimately, male infertility (Figure 7). Our study enriches the knowledge regarding the role of PK2 in inflammatory diseases and provides a new viewpoint on the mechanisms underlying inflammation-associated male infertility. Moreover, these data suggest that PK2 is a potential immunomodulatory biomarker for orchitis and a novel anti-inflammatory therapeutic target for treating male infertility.

## Data Availability

All datasets generated for this study are included in the manuscript and/or the Supplementary Files.

## Ethics Statement

This study was performed in strict accordance with the approved guidelines from the Institutional Animal Care and Use Committee of Tongji Medical College, Huazhong University of Science and Technology.

## Author Contributions

YL and KZ designed research studies, conducted experiments, analyzed data, and drafted the manuscript. YS conducted rat model establishment, cell isolation, and propagation of bacteria. TZ assisted in western blot and immunofluorescence of PK2 detection. HZ conducted animal execution and semen analysis. JW and WW conducted collection of testicular interstitial fluid and propagation of bacteria. CL and ZH provided intellectual input into planning of experiments and contributed to the writing of the manuscript.

### Conflict of Interest Statement

The authors declare that the research was conducted in the absence of any commercial or financial relationships that could be construed as a potential conflict of interest.
