# Supplementary material for: Activation of the NLRP3 Inflammasome Pathway by Prokineticin 2 in Testicular Macrophages of Uropathogenic Escherichia coli- Induced Orchitis
Source: Front Immunol. 2019 Aug 14;10:1872. doi: 10.3389/fimmu.2019.01872 (PMC6702272; doi:10.3389/fimmu.2019.01872)
Supplement: Supplementary file 1 [file Data_Sheet_1.doc]

**Supplementary materials**

**
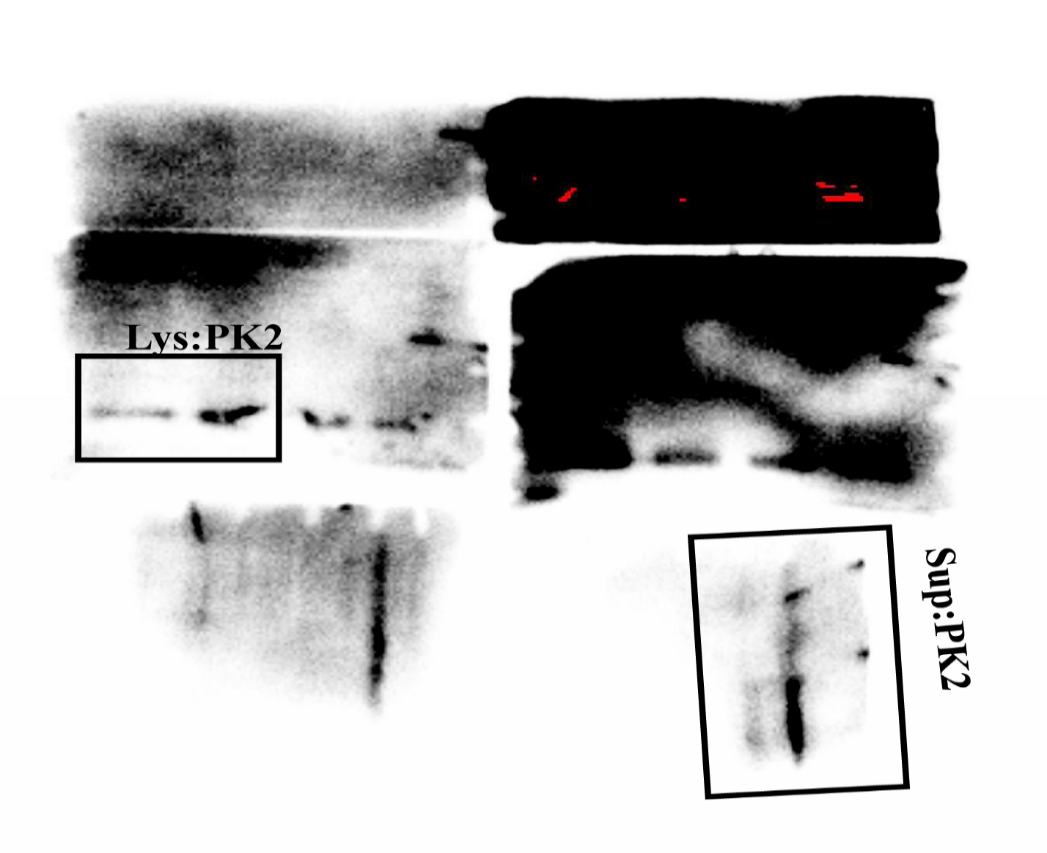
**

**Supplementary Figure 1**. Bands were detected using anti-PK2 antibody in the testicular macrophages and the supernatants in Figure 1. Lys: cell lysates, Sup: supernatants.


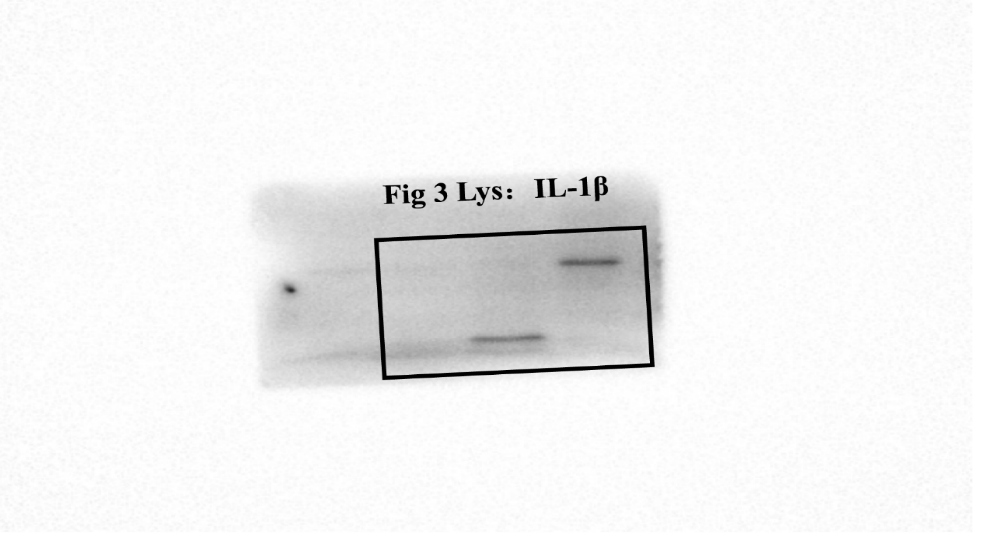


**Supplementary Figure 2**. Bands were detected using anti-IL-1β antibody in the testicular macrophages in Figure 3. Lys: cell lysates.


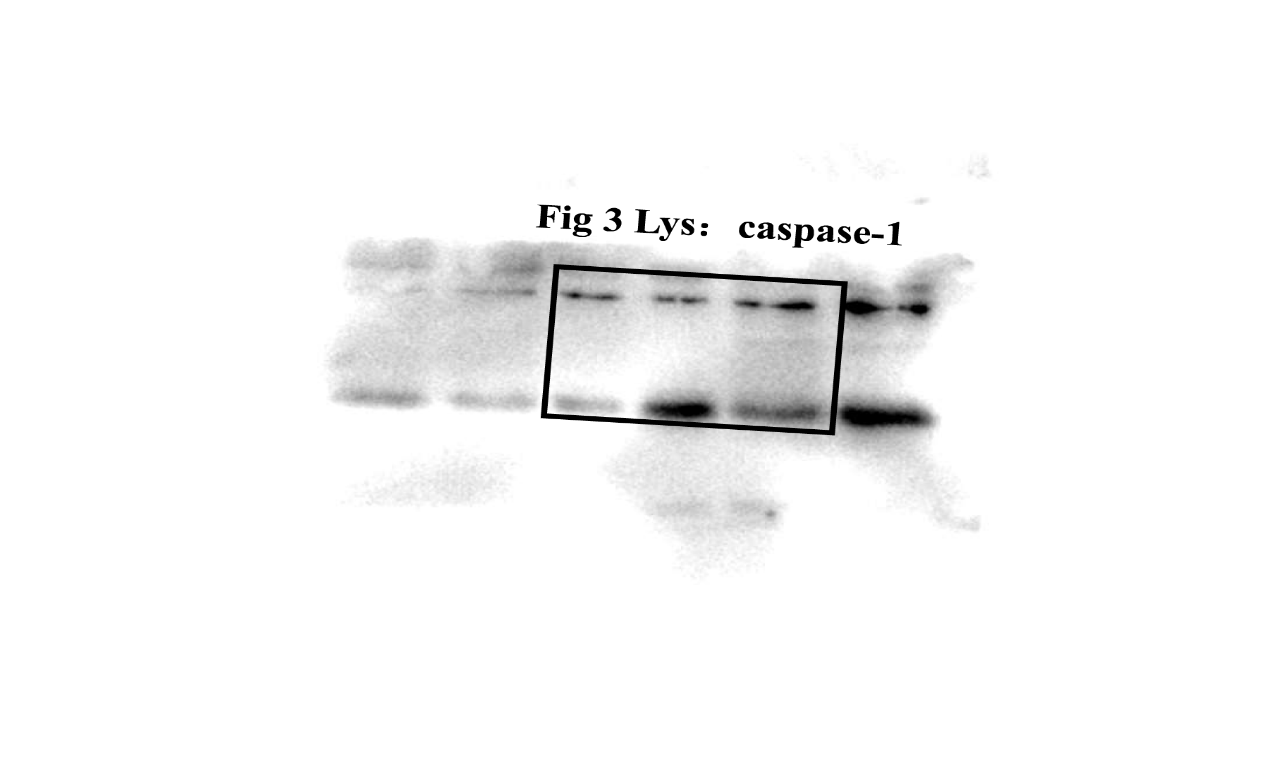


**Supplementary Figure 3**. Bands were detected using anti-caspase-1 antibody in the testicular macrophages in Figure 3. Lys: cell lysates.


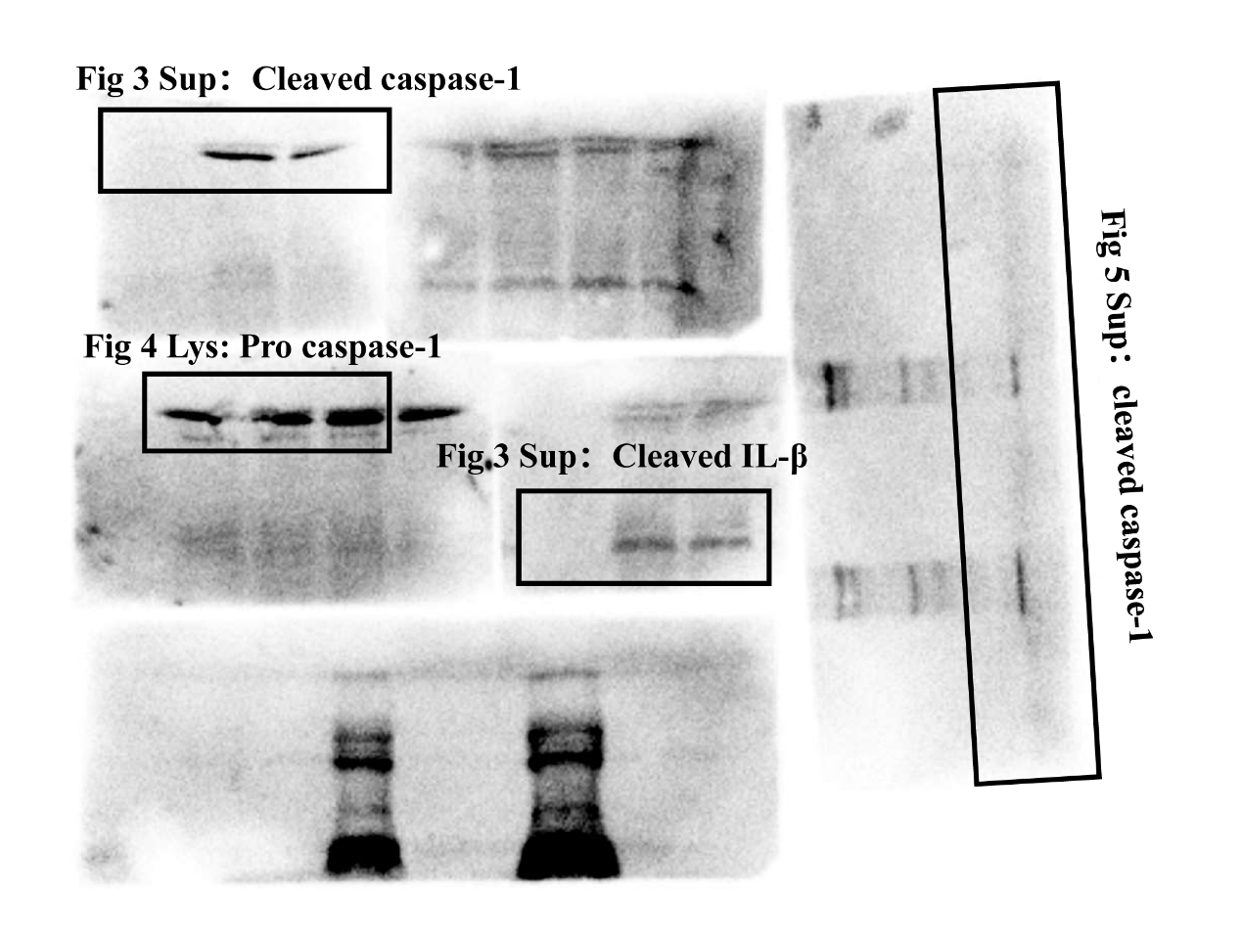


**Supplementary Figure 4**. Bands were detected using anti-caspase-1 antibody and anti-IL-1β antibody in the testicular macrophages and the supernatants in Figure 3, Figure 4 and Figure 5. Lys: cell lysates, Sup: supernatants.


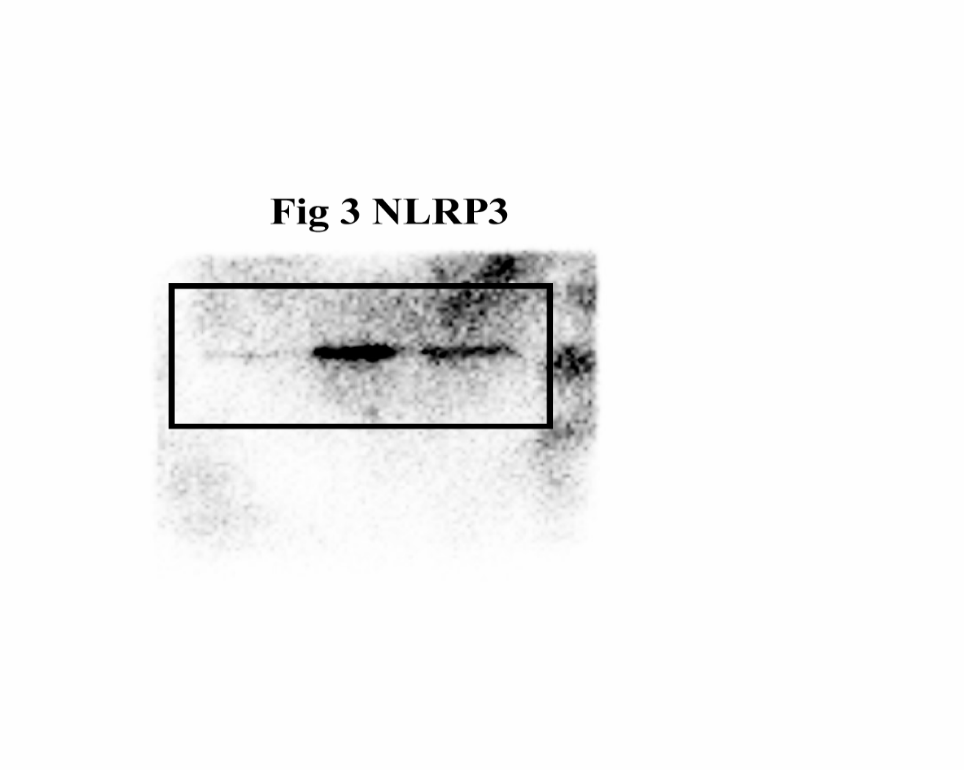


**Supplementary Figure 5**. Bands were detected using anti-NLRP3 antibody in the testicular macrophages in Figure 3. Lys: cell lysates.


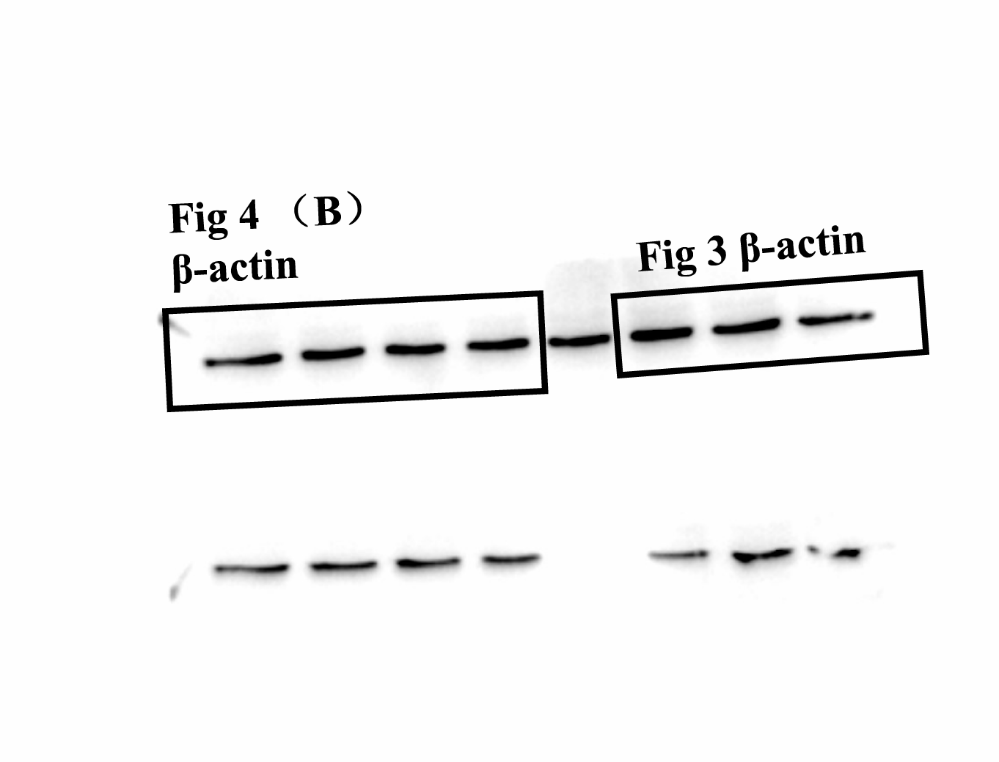


**Supplementary Figure 6**. Bands were detected using anti-β-actin antibody in the testicular macrophages in Figure 3 and Figure 4 (B).


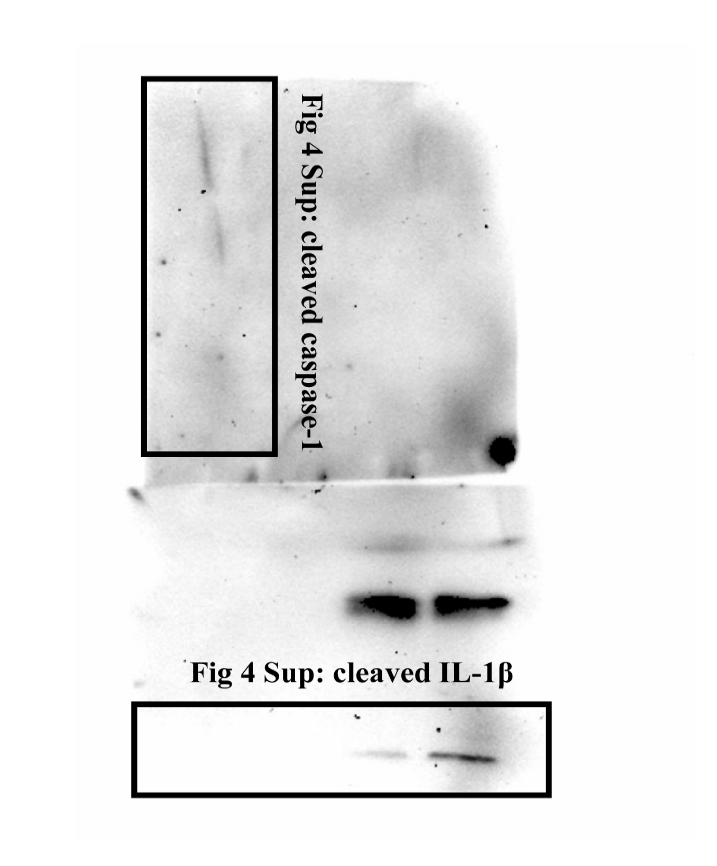


**Supplementary Figure 7**. Bands were detected using anti-caspase-1 antibody and IL-1β antibody in the supernatants in Figure 4. Sup: supernatants.


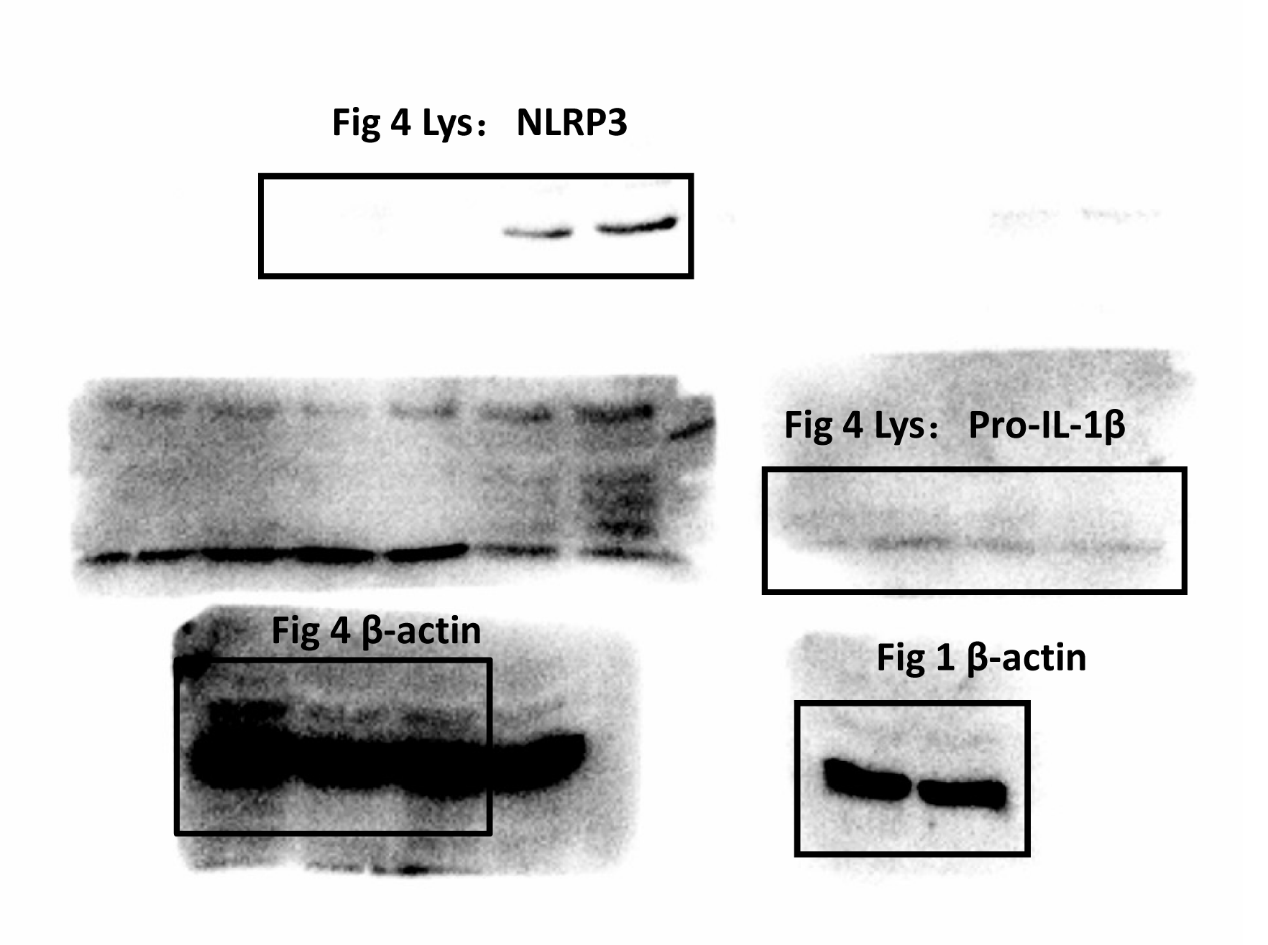


**Supplementary Figure 8**. Bands were detected using anti-caspase-1 antibody, anti-IL-1β antibody, anti-NLRP3 antibody and anti-β-actin in the testicular macrophages in Figure 1 and Figure 4. Lys: cell lysates.


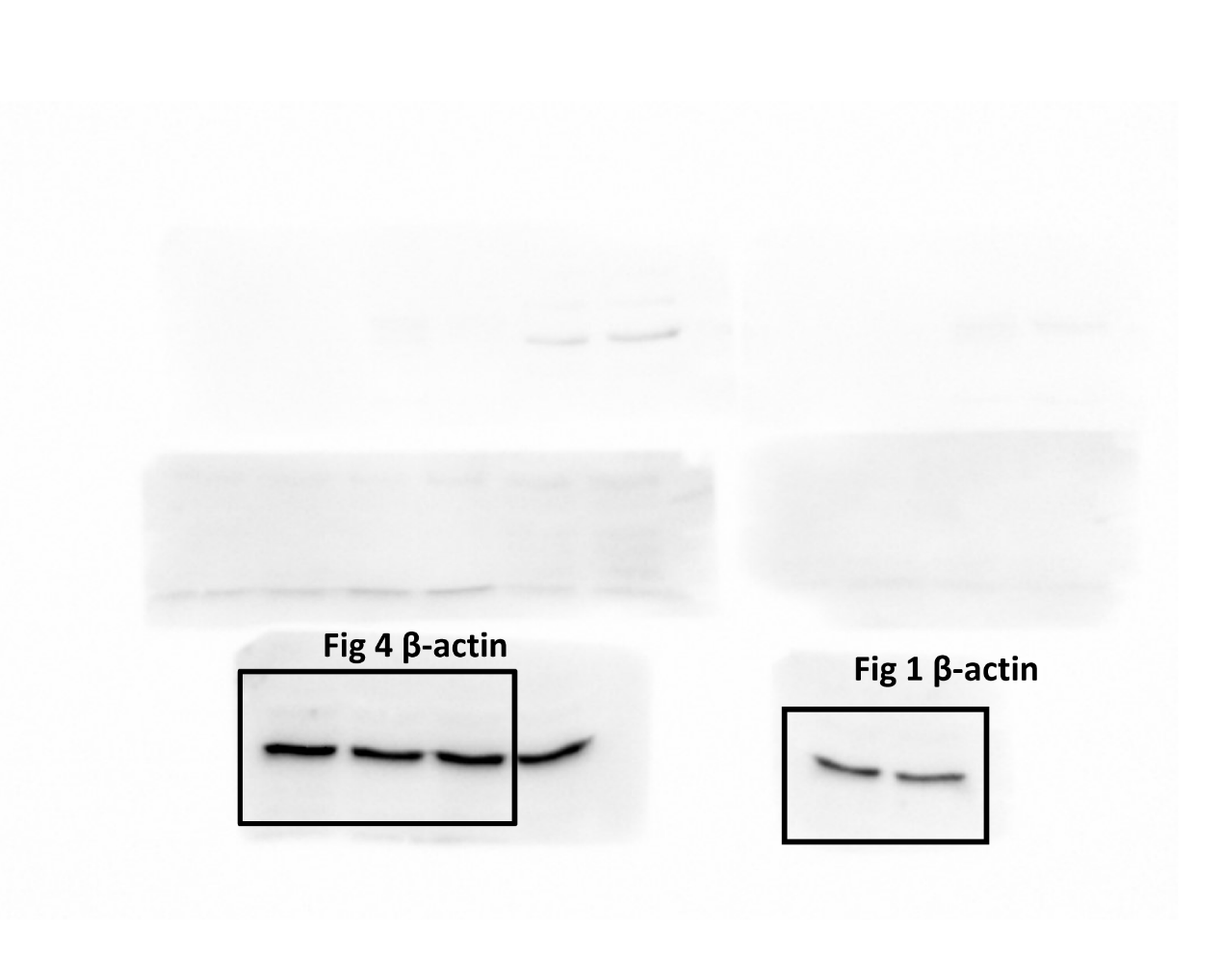


**Supplementary Figure 9**. Bands were detected using anti-β-actin in the testicular macrophages in Figure 1 and Figure 4 (Another exposure time to Supplementary Figure 8).


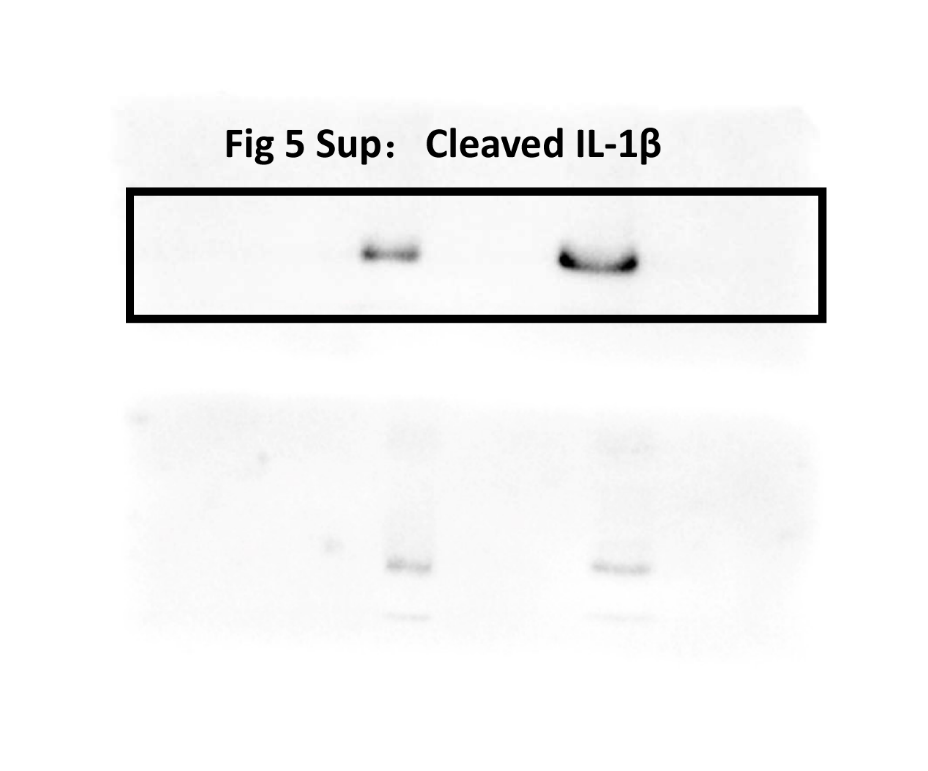


**Supplementary Figure 10**. Bands were detected using anti-IL-1β antibody in the supernatants in Figure 5. Sup: supernatants.


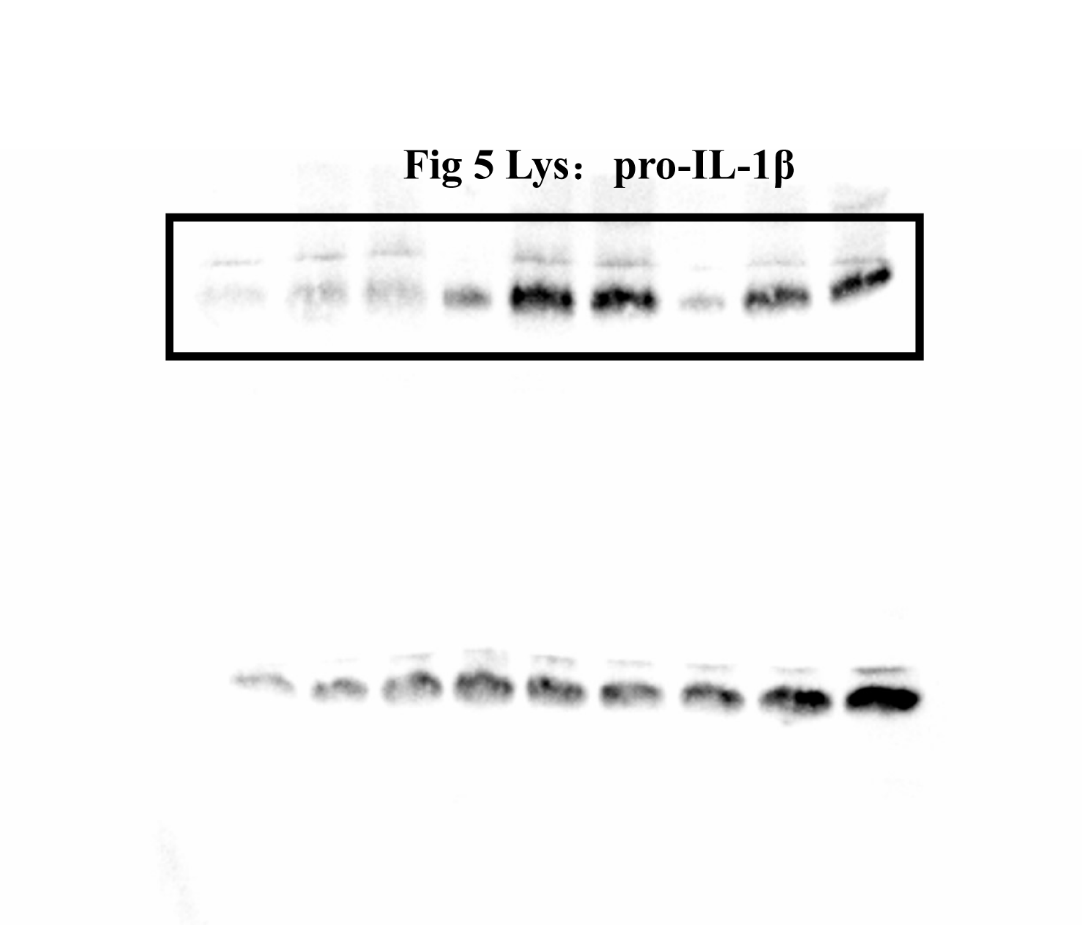


**Supplementary Figure 11**
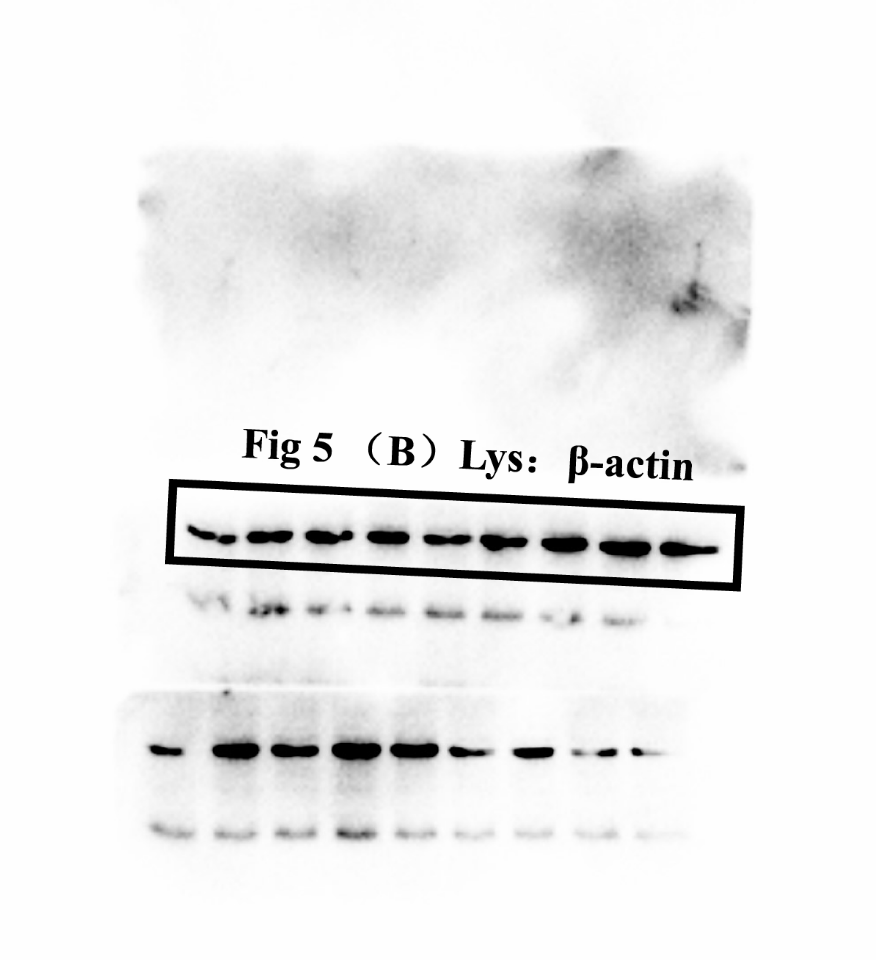
. Bands were detected using anti-IL-1β antibody in the testicular macrophage in Figure 5. Lys: cell lysates.

**Supplementary Figure 12**. Bands were detected using anti-β-actin antibody in the testicular macrophages in Figure 5 (B).


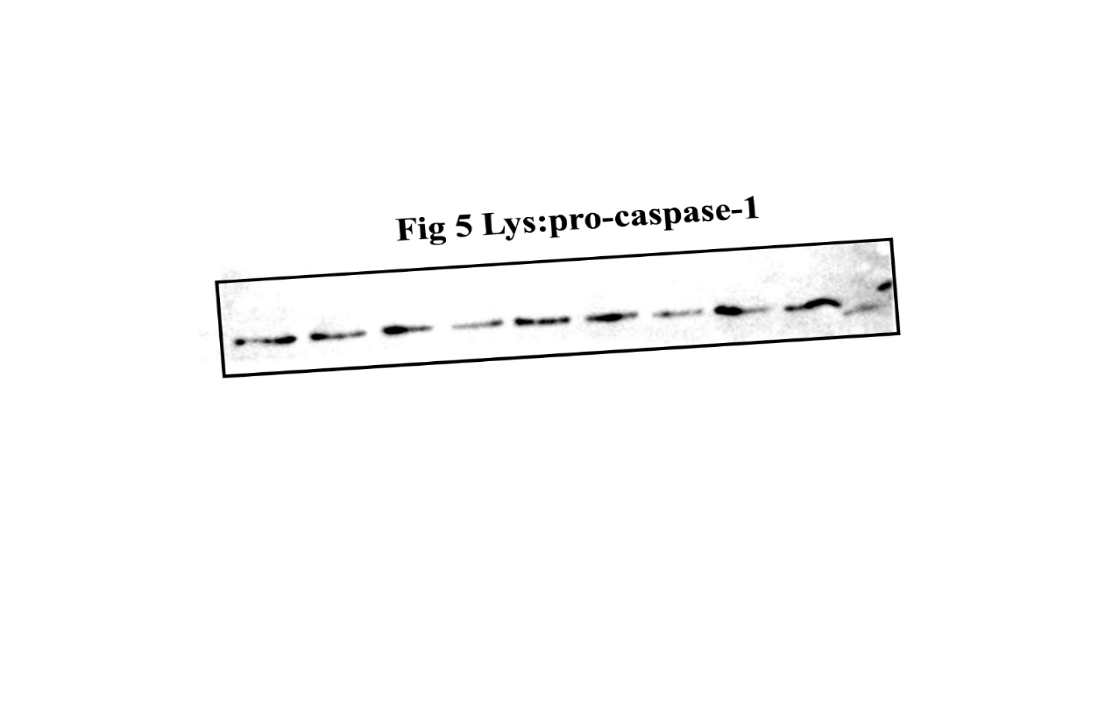


**Supplementary Figure 13**. Bands were detected using anti-caspase-1 antibody in the testicular macrophage in Figure 5. Lys: cell lysates.


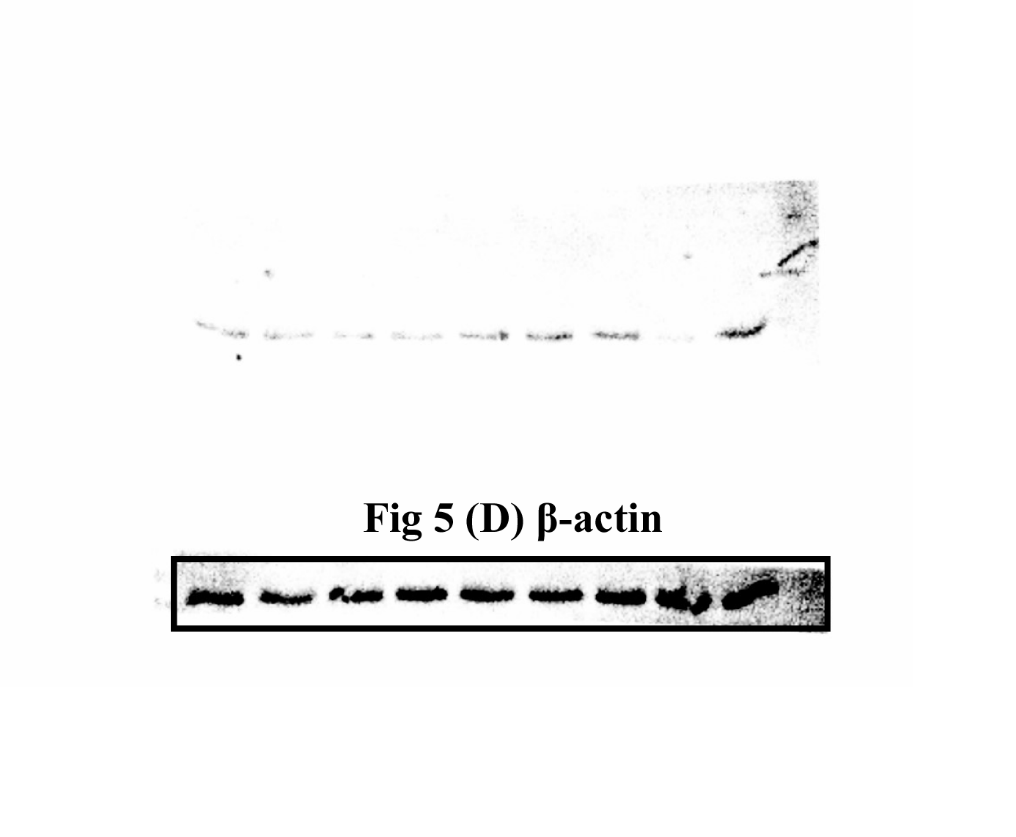


**Supplementary Figure 14**. Bands were detected using anti-β-actin antibody in testicular macrophages in Figure 5 (D).
